# Exploring the Bioactive Potential of Moroccan Lemon Grass (*Cymbopogon citratus* L.): Investigations on Molecular Weight Distribution and Antioxidant and Antimicrobial Potentials

**DOI:** 10.3390/molecules29173982

**Published:** 2024-08-23

**Authors:** Ahmed Tazi, Sara El Moujahed, Noura Jaouad, Hamza Saghrouchni, Ibrahim Al-Ashkar, Liyun Liu, Faouzi Errachidi

**Affiliations:** 1 Laboratory of Functional Ecology and Environmental Engineering, Faculty of Sciences and Technology, Sidi Mohamed Ben Abdellah University, Fez 30000, Morocco; sara.elmoujahed@gmail.com (S.E.M.); errachidifaouzi@yahoo.fr (F.E.); 2 Laboratory of Engineering, Electrochemistry, Modeling and Environment (LIEME), Faculty of Sciences Dhar Lmehraz, Sidi Mohamed Ben Abdellah University, Fez 30000, Morocco; nourajaou@gmail.com; 3 Department of Biotechnology, Institute of Natural and Applied Sciences, Cukurova University, Balcalı, 01330 Adana, Turkey; 4 Plant Production Department, College of Food and Agriculture Sciences, King Saud University, P.O. Box 2460, Riyadh 11451, Saudi Arabia; ialashkar@ksu.edu.sa; 5 Graduate School of Engineering, Osaka University, 2-1 Yamadaoka, Suita, Osaka 565-0871, Japan; liuliyun79419@163.com

**Keywords:** lemon grass, bioactive molecules, antioxidant activity, antimicrobial activity

## Abstract

Research on lemon grass (*Cymbopogon citratus* L.) revealed a variety of active molecules and examined their biological characteristics. However, most of these studies were conducted on wild varieties, while cultivated plants were addressed less. This study aimed to characterize the biomolecules and biological activities of lemon grass growing under North African conditions in Morocco. Phenolic compound profiles of aqueous (AE), ethanol (EE), and methanol (ME) extracts and their fractions were obtained with steric exclusion chromatography on Sephadex G50 gel and identified by LC-MS/MS. Then, total polyphenols (TPC), flavonoids (TFC), and antioxidant activities (FRAP: scavenging value and TAC: Total Antioxidant Capacity) of the fraction were evaluated, as well as the antimicrobial activity. The obtained results showed that the ME contained eight major compounds (i.e., apigenine-7-O-rutinoside and myricitine-3-O-rutinoside). The AE showed the presence of five molecules (i.e., kaempferol-3-O-glucuronide), while EE showed the presence of three molecules (i.e., quercetine-3-O-rutinoside). Regarding the chemical characterization, the highest value of total phenolic content (TPC) was obtained in AE (25) (4.60 ± 0.29 mg/g), and the highest value of total flavonoid content (TFC) was obtained in ME (29) (0.7 ± 0.08 mg/g). Concerning the antioxidant activity, the highest FRAP was obtained in ME (29) (97.89%), and the highest total antioxidant capacity (TAC) was obtained in ME (29) (89.89%). Correlation between FRAP, TPC, and TFC was noted only in fractions of AE and ME. All tested extracts of *C. citratus* and their fractions showed a significant antimicrobial effect. The lowest minimum inhibitory concentration (MIC) was recorded for ME against *E. coli*. Extracts’ biological activities and their fractions were governed by their active molecules. These data are new and clarify a novel aspect of bioactive molecules in the extracts of cultivated *C. citratus*. Equally, throughout this research, we clarified the relationship between identified molecules and their biological properties, including antioxidant and anti-microbial activities, which is new for the study area. This study is suggested as a reference for comparative studies and other assays of other biological activities for the study plant.

## 1. Introduction

Plants are a significant source of medicinal agents since they contain a wide variety of active ingredients (i.e., bioactive molecules) with significant therapeutic effects [1,2]. Because they are easily accessible and have a lower harmful effect on the receiver than synthetic pharmaceuticals. So, plant-based medications have been utilized to treat a wide range of disorders throughout the world since antiquity [1,3,4,5]. Herbal pharmaceutical usage is expanding quickly and makes up a large portion of the global drug market [6]. For their fundamental health needs, more than 75% of the world’s population relies on medicinal herbs [7]. Because it has no side effects, it became a well-liked alternative to synthetic medicine [8,9].

Numerous chemical substances classified as primary and secondary metabolites are produced by plants [10]. While secondary metabolites have a variety of medical uses, primary metabolites are directly involved in growth and development [11]. There is a broad range of secondary metabolites, such as alkaloids, flavonoids, tannins, cardiac glycosides, saponins, terpenoids, etc. [12]. Each of these serves a certain purpose and offers health benefits. As a result, pharmaceutical and cosmetic sectors use them as primary materials [13,14]. On the other hand, these bioactive molecules are extracted from essential oils, extracts, and infusions of medicinal plants [15,16,17]. However, the bioactive molecules differ depending on the extraction method [18], extraction solvent [17], plant material [19], and growing conditions of plants [20]. Equally, bioactive molecules differ depending on their molecular weight [21] and polarity [22], which influences their extraction and interaction with extraction solvents [23].

Lemon grass (*Cymbopogon citratus*) is a member of the Poaceae family and the genus [24,25]. A tall, monocotyledonous, scented perennial plant, *Cymbopogon* has narrow green leaves with sharp edges and pointed tips [26]. *C. citratus* plant leaves and other parts are utilized in food, cosmetics, and pharmaceutical products [27,28]. One of the most significant therapeutic plants, lemon grass, has several uses in conventional medicine [29,30]. Additionally, it can be used to treat HIV side effects, including secondary bacterial infections [1,31]. Due to the presence of different secondary metabolites, lemongrass has historically been used to treat a variety of illnesses [29]. It has also been used to treat cough, fever, elephantiasis, flu, malaria, leprosy, and other digestive difficulties [32]. It has also been observed that lemon grass possesses antimicrobial properties against a variety of bacteria, fungi, and protozoa [33]. It was tested against *Staphylococcus aureus, Candida* pathogens species [33], and *Salmonella enterica* [34].

*C. citratus* biological properties are related to its biochemical constituents [35,36,37]. The analysis of essential oils extracted from lemon grass leaves revealed a huge chemical diversity of chemical components, counting 72 bioactive molecules [38,39,40]. The major elements were geranial, neral, geraniol, limonene, and β-myrcene [39]. However, these bioactive constituents vary depending on climate conditions and geographical areas of analyzed plants, as well as depending on tested parts of lemon grass [37,39,40]. For example, Tazi et al. (2024) [37] demonstrated a significant qualitative and quantitative variation of bioactive molecules from the extracts of *C. citratus* among samples growing in Asia, Europe, South America, and Africa. In parallel, current studies showed important quantities of total phenolic compounds and flavonoid contents and antioxidant activity [41,42], which are suggested to increase lemon grass’s biological activities’ effectiveness.

In Morocco, investigations concerning the chemical composition and biological properties of *C. citratus* are fragmentary [43,44,45]. Only one study addressed the composition of the chemical *C. citratus* essential oils [44]. Similarly, one study tested the EOs inhibition effect against *Aggregatibacter actinomycetemcomitans* virulent strains [43]. Therefore, more investigations are needed to determine the antioxidant activities and phenolic and flavonoid contents of varieties of *C. citratus* that are grown in Morocco so far from its native area (Asia). Its introduction to Morocco constitutes a challenge for farmers who want to promote this high-potential plant. Our contribution aims to characterize this plant acclimatized to Morocco and inquire about its adaptation to the new climate.

In this context, this study aimed to establish phenolic compounds molecular profiles of the dominating aqueous (AE), ethanol (EE), and methanol (ME) extracts of lemon grass and their fractions with steric exclusion chromatography on Sephadex G50 and identification by Liquid Chromatography coupled to tandem Mass Spectrometry (LC-MS/MS). Then, we investigated total polyphenols, flavonoids, and antioxidant activities (FRAP and TAC) variations in each chromatographic fraction. Equally, we tested their inhibitory effects against Gram-negative bacteria (*Escherichia coli, Pseudomonas aeruginosa*), Gram-positive bacteria (*Bacillus cerieus*, *Staphylococcus aureus*), and fungi (*Candida tropicalis* and *Saccharomyces cerevisiae*). Besides, we have evaluated the minimum inhibitory concentration (MIC) and minimum bactericide and fungicide concentration (MBC). The results of these studies are suggested to present new data on bioactive molecules and their effects on the biological properties of the extracts from *C. citratus* growing under North African conditions in Morocco.

## 2. Results and Discussion

### 2.1. Yield of Extracts

**Figure 1:**
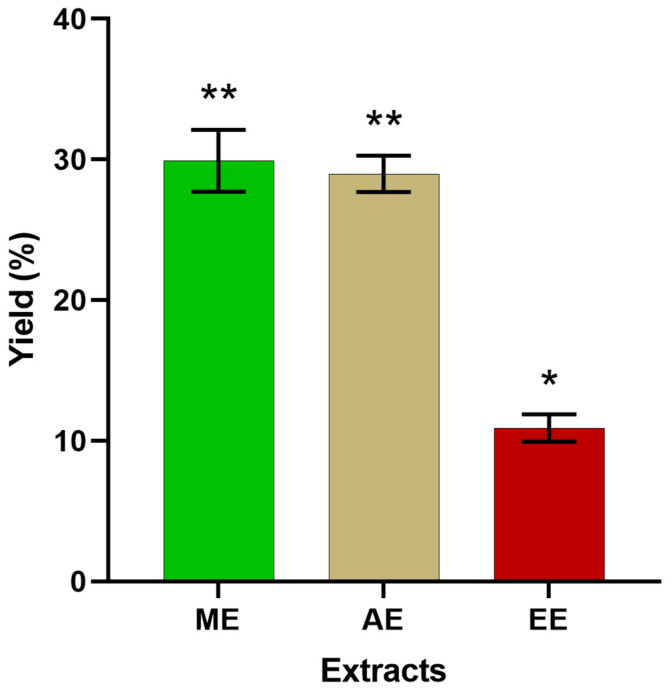
Yields extraction of solvents used in phenolic compounds extraction from *C. citratus*. ME—methanolic extract, AE—aqueous extract, and EE—ethanolic extract. (* Denotes statistical difference between extracts at *p* < 0.05; ** > *).

The extract yield was variable depending on the type of solvent polarity. The highest yield (29.92%) was obtained with methanol, the medium yield (28.98%) was recorded with water, and the lowest yield (10.9%) was obtained with ethanol (Figure 1). Currently, Olukunle and Adenola [46] investigated the yields of extracts from wild *C. citratus* in Nigeria. These authors used three solvents, including water, methanol, and ethanol. In the results, the highest yield (6.67 ± 0.11%) was obtained in water, followed by methanol (4.15 ± 0.08%), and the lowest value (3.69 ± 0.12%) was noted for ethanol. These cited values are inferior when compared to the results of this experiment, ranging between 10.9 and 29.92%. The recorded difference for tested extracts is suggested to be ruled by the polarity and affinity of each extract. Water and ethanol are known for their polarity toward phenolic compounds [47,48], which explains their higher yield.

### 2.2. The Polymerization Profiles of Crude Extracts

**Figure 2:**
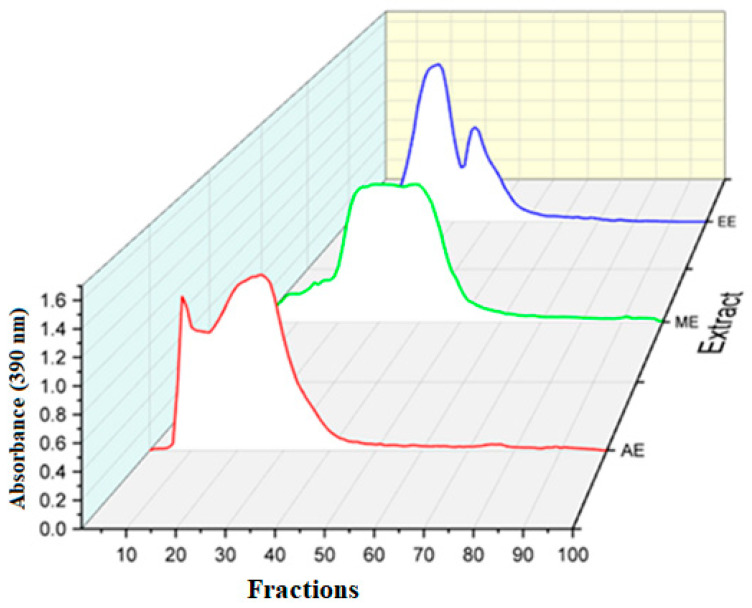
Molecular weight distribution patterns of phenolic compounds extracted from lemon grass (AE—aqueous extract, ME—methanolic extract, and EE—ethanolic extract).

**Figure 3:**
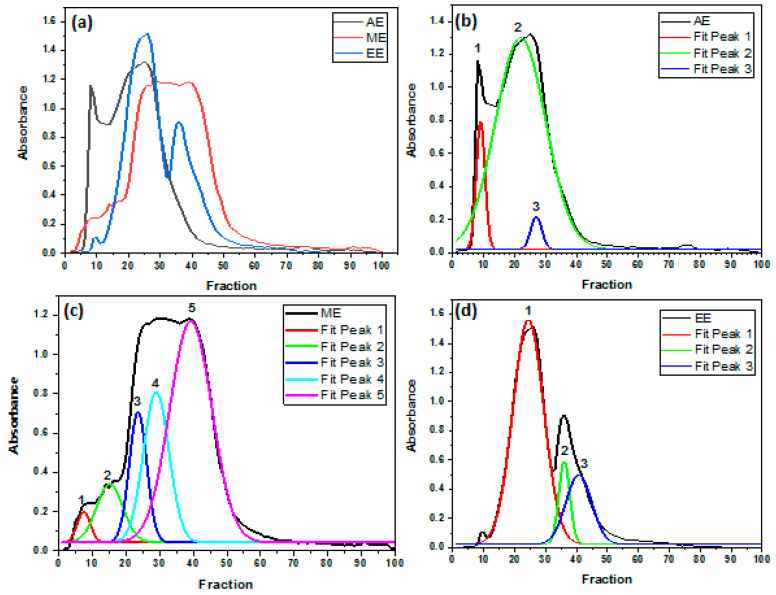
Elution profiles after deconvolutions: (**a**) all extracts, (**b**) aqueous extract—AE; (**c**) methanolic extract—ME; (**d**) ethanolic extract—EE.

Figure 2 and Figure 3 show the molecular weight distribution patterns of each *C. citratus* crude extract to better understand the molecular sizes of active ingredients and their potential usage in future applications.

Figure 2 illustrates the selectivity of solvent toward phenolic compounds’ molecular weight. As reported previously by Ponnuchamy et al. [49], the smaller the fraction number, the higher the molecular weight and vice versa. Indeed, water showed a high selectivity for high molecular weight, which is revealed from fraction 7 to fraction 20. Further, methanol shows a high selectivity for medium phenolic compounds’ molecular weight, eluted from fractions 20 to 40. In contrast, ethanol shows high selectivity for low molecular weight phenolic compounds eluted from fractions 40 to 60. In our case, it may be said that various extracts under study show variations in phenolic content’s molecular distributions, both qualitatively and quantitatively. Overall, the affinity of the solvent used for *C. citratus* extraction, face to high molecular weight, increases proportionally with polarity degree. However, the results obtained were not in line with a previous study by Soliman et al., where it was found that aqueous extract is more selective than methanolic extract in compounds with a low molecular weight [50].

In Figure 3b, AE showed three main molecular weights, 1, 2, and 3, located at fractions 9, 22, and 27, respectively. Peak 1 represents the highest polymerization degree among eluted profiles. Previous studies corroborated these results and reported that the high molecular weight of extracts correlates well with low yields [51]. In Figure 3c, the ME extract was reduced to five weights located at fractions 8, 15, 23, 29, and 39. Thus, methanol as a solvent was appropriate for both low and high-molecular-weight phenolic compounds. In Figure 3d, the EE was reduced to three main peaks deconvoluted corresponding to fractions 24, 36, and 41, respectively. Ethanol was suitable for monomer extraction, where peaks 2 and 3, with their small size, were delayed between Sephadex gel beads, leading to their slow migration. Otherwise, ethanol was reported previously as a suitable solvent for the extraction of low molecular weight phenolic compounds as flavonoids and high molecular weight as tannins [52,53,54].

### 2.3. Chemical Compounds

The results of LC-MS-MS analysis are presented in Table 1. Recorded results showed that the extracts of lemongrass are rich in phytochemicals. However, the bioactive molecules are variable among the extracts. Methanolic extracts demonstrated eight biomolecules differentiated by their molecular weight. Apigenin-7-O-rutinoside showed the highest molecular weight (585.2 *m*/*z*), followed by myricitine-3-O-rutinoside (565.21 *m*/*z*) and 11beta,17alpha,21-Trihydroxy-4-pregnene-3,20-dione 21-caprylate (487.31 *m*/*z*). In contrast, luteoline-7-O-rutinoside and quercetin-3-O-arabinoside showed inferior molecular weights (393.22 and 379.16 *m*/*z*, respectively). Further, five molecules were identified in water extracts; kaempferol-3-O-glucuronide showed the highest molecular weight (487.306 *m*/*z*), while myricetin showed the lowest molecular weight (377.08 *m*/*z*). In ethanol extract, only three molecules were recorded. Further, quercetin-3-O-rutinoside showed the highest molecular weight (665.166 *m*/*z*), while myricetine-3-O-glucuronide showed the lowest molecular weight (497.3351 *m*/*z*).

In the last decade, Roriz et al. [55] conducted a phytochemical study to investigate the active molecules and antioxidant activity in *C. citratus*, *Gomphrena globose*, and *Pterospartum tridentatum*. The authors used methanolic extracts and recorded bioactive molecules via HPLC coupled with spectrophotometry (HPLC, Hewlett-Packard 1100, LA, CA USA). Double online detection was carried out in the diode array detector (DAD) using 280 nm and 370 nm. The obtained results showed 18 phenolic compounds in *C. citratus*, compared to 21 in *P. tridentatum* and 27 in *G. globose*. Among the recorded molecules in *C. citratus*, the authors mentioned luteolin 6-C-pentoside with a molecular weight of 399 *m*/*z* and apigenin 6-C-pentosyl-8-C-hexoside with a molecular weight of 545 *m*/*z*. The recorded molecules are very close to the molecules recorded in our sample’s methanol extract. The slight difference recorded in terms of fragments between our results and those recorded by Roriz et al. [55] is suggested to be related to the methods used. In our case, we used HPLC LC-MS-MS, which is more sensitive compared to HPLC coupled with spectrophotometry. On the other hand, the difference in chemical compounds among the extracts is suggested to be governed by solvent polarity. For example, many studies have confirmed the capacity of methanol solvents to extract a wide range of compounds, including phenols and flavonoids, compared to water and ethanol [56,57,58], which is in agreement with our results. 

In our case, two compounds, quercetin and myricetin, were recorded in all the extracts, but their distribution varied, which explains the effect of each solvent polarity. The higher number of molecules in methanol extract is governed by three factors: (i) methanol is a polar solvent, which means it has a partial electrical charge, which allows methanol to bind to flavonoids, which are also electrically charged; (ii) methanol extraction is with low ionic strength (is not very acidic or basic) [59], which is preferable for flavonoids extraction [60], as it preserves their biochemical properties [61]; and (iii) methanol extraction can be carried out at room temperature or at higher temperature [62], which is preferable to preserve the biochemical properties of flavonoids. Generally, methanol is a good solvent for fat-soluble compounds, such as non-glycosylated flavonoids and terpenoids [63]. In contrast, water is a good solvent for water-soluble compounds, such as flavonoid glucuronides [64]. Further, ethanol is a good solvent for a combination of water-soluble and fat-soluble compounds.

Different studies have addressed the chemical compounds in lemon grass due to its biological properties [65,66,67,68,69]. These investigations targeted the essential oils and extracts to identify the molecules responsible for the biological effects of this plant. For example, Kabotso et al. [70] addressed the chemical compounds in the extracts of lemon grass to explain their antimicrobial activities against resistant *Staphylococcus aureus*. In total, eight chemical compounds dominated by two isomers, neral and geranial of citral, and the acetate geranyl acetate, were identified in both water and ethanol extracts and the essential oil of lemon grass. These results are in agreement with our results in terms of the number of compounds, while the type of molecules is significantly different compared to our results. The diversity of chemical compounds in our samples is suggested to promote robust biological activities in the extracts [71,72]. Further, the diversity of bioactive molecules in our extracts is suggested to profit from the single and synergetic effects of each chemical compound [73,74]. On the other hand, our results showed a significant difference in chemical compounds depending on the used solvent and fraction. In addition, a higher number of compounds was identified in the methanol extract compared to ethanol and water. This difference in chemical compounds among the extracts is suggested to be governed by the polarity of each solvent. For example, many studies have confirmed the capacity of methanol solvents to extract a wide range of compounds, including phenols and flavonoids, compared to water and ethanol [56,57], which is in agreement with our results.

To increase the depth of recorded bioactive compounds, we coupled the results obtained by LC-MS/MS and chromatograms of Sephadex. The chemical analysis showed that the methanolic extract comes first with eight molecules, some of which are superimposed in the Sephadex chromatogram. The first peak was shared between the two molecular weights, 585.20 *m*/*z* and 565.21 *m*/*z*, with different retention times. The second peak corresponded to a molecule at 487.31 MW. The third peak was also shared between the molecules at PM 457.19 *m*/*z* and 431.23 *m*/*z*. The fourth 417.21 and the fifth peak corresponded to two molecules with molecular weights of 393.22 m/z and 379.16 *m*/*z*. Furthermore, two superpositions having a molecular weight of 487.306 (100% superimposed) at different retention times, 5.70 and 6.06 min, were recorded in the extract of distilled water. These correspond to the first peak of the Sephadex chromatogram. The second peak was shared between 477.08 *m*/*z* and 457.19 *m*/*z* and the third peak corresponds to molecular weight 377.08 *m*/*z*. The ethanolic extract Sephadex G50 chromatogram and the LC-MS-MS analysis coincide well. The chromatogram showed three unique molecules that give rise to three peaks, with molecular weights of 665.16 for the first peak, 545.13 *m*/*z* for the second, and 497.33 *m*/*z* for the third.

Eight active molecules were present in the methanolic extract, which explains its biological activity. Five molecules are present in distilled water from those molecules, and three active molecules are present in the ethanolic extract, which rounds out the list. The distribution of molecular weights according to the polymer-forming phenolic fractions, with the least amount of monomeric phenolic chemicals from fraction 70, is revealed by spectra comprehensive examination. Peak’s frequency changes with the type of solvent, and we observed that solvent polarity affects how many peaks there are, with a minimal intersection. As we mentioned above, the difference in molecules among the extracts is suggested to be related to the polarity of each solvent.

### 2.4. Total Phenolic Compounds Determination

The TPC quantity recorded in tested extracts is presented in Table 2. TPC quantities in *C. citratus* were variable depending on the type of extract and fraction. In AE, the quantity of TPC was significantly variable among fractions, and the highest quantity was recorded in fraction 25. TPC value in pick 16 comes in the second rank, followed by pick 8, while the lowest values were recorded in pick 58 and pick 75, respectively. In EE, TPC was significantly superior in pick 26 when compared to 36 (*p* < 0.001). In ME, TPC was significantly superior in pick 29 when compared to pick 23.

The total phenolic content is widely investigated in different parts of lemongrass, including leaves, flowers, and roots [42,75]. In results, investigations showed that the quantity of phenolic compound varies depending on the part of *C. citratus* used, extraction methods, solvent, geographical area, etc. Unuigbe et al. [76] evaluated phenolic compound contents in crude methanol extract and its fractions (*n*-hexane, ethyl acetate, and chloroform) of powdered leaves of *C. citratus* using the Folin–Ciocalteu and aluminum chloride methods. The ethyl acetate fraction had the highest phenolic content (172.5 mg GAE/g extract) among extracts and fractions. This was followed by chloroform fraction (160.0 mg GAE/g extract), methanol extract (132.5 mg GAE/g extract), and *n*-hexane fraction (104.0 mg GAE/g extract) [76]. In another study, Godwin et al. [77] recorded values of total phenolic compound activity in cold and hot percolations ranging from 1.3 to 4.7 mg and 2.6 to 7.3 mg of gallic acid equivalents (GAE)/g (dw), respectively. In Malaysia, Sin Yen Sah et al. [78] evaluated the quantity of phenolic compounds in commercialized fresh lemon grass using the Folin–Ciocalteu method. In the results, the TPC value of 67 mg GAE/g, and was positively correlated with the antioxidant activities of lemon grass leaf extract assessed by FRAP (r = 0.995). Irfan et al. [42] used maceration and sonication techniques to investigate lemon grass collected in Islamabad, Pakistan. Results showed that acetone was the most effective solvent, while ethanol showed the lowest phenolic compound content. The highest total phenolic content (55.2 mg GAE/g of extract) was extracted with acetone solvent at a 50% concentration, whereas with 70% ethanol, they obtained the lowest quantity of polyphenols (32.9 mg GAE/g of extract). Meanwhile, the sonication technique results showed that with 50% ethanol, the maximum polyphenols were extracted (61.2 mg GAE/g of extract), while 70% acetone extracted the minimum quantity of phenolic compounds (50.9 mg GAE/g of extract). Sepahpour et al. [75] conducted a comparative analysis to evaluate phenolic compounds in lemon grass, turmeric (*Curcuma longa*), torch ginger (*Etlingera elatior*), and curry leaf (*Murraya koenigii*) using different solvent extraction systems. 

The quantity of TPC in the extracts indicated a wide variation. Turmeric acetone extract exhibited the highest quantity of phenolic compounds (221.7 mg gallic acid equivalent (GAE)/g of freeze-dried crude extract (CE)), while lemon grass water extract demonstrated the lowest amount of total phenolic compounds (1.2 mg GAE/g CE). TPC values in our study vary between 0.11 ± 0.03 and 4.60 ± 0.29 mg GAE/g of extract, which is in agreement with the cited results. Moreover, our investigations showed a great variation in TPC depending on the type of extract and fraction, which is the first of its kind for this plant. In our case, the maximum TPC was obtained in fraction 25 of aqueous extract. A higher value of phenolic compound was recorded in fractions of ME and AE compared to other fractions, and this is suggested to be due to the polar nature of these components (see Figure 2). Phenolic compounds are generally polar, and solvents appear to play a significant role in their extraction, so polar solvents tend to contain more of these components than less polar or non-polar solvents [76]. 

In our case, variation in TPC values is also suggested to be governed by the size of separated molecules in each extract and fraction. The higher value of TPC in fraction 25 of AE is due to the dominance of molecules characterized by higher molecular weight. The second highest value of TPC in fraction 29 of ME is suggested to be related to the presence of molecules characterized by medium molecular weight (see curve 4, graph b, Figure 3). In contrast, the dominance of molecules characterized by low molecular weight is suggested to explain the lower values of TPC in EE fractions (see curve 1, graph c, Figure 3). Similar results were currently demonstrated in melanoidin fractions derived from two different types of cocoa beans by UHPLC-DAD-ESI-HR-MSn [79,80].

### 2.5. Total Flavonoid Content (TFC) Determination

Recorded quantities of TFC in tested extracts are presented in Table 3. TFC quantities in *C. citratus* were variable depending on the type of extract and fraction. In AE, TFC quantity was significantly variable among fractions, and the highest quantity was recorded in fraction 8, followed by 25. In contrast, the lowest TFC value was recorded in fraction 16. In EE, TFC was significantly superior in fraction 26 compared to fraction 36 (*p* < 0.001). In ME, TFC was significantly superior in fraction 29 when compared to fraction 23.

Lemon grass is known for its richness in flavonoid contents [75,76,77,81]. However, as for TPC, flavonoid quantity varies depending on the used part of C. citratus, extraction methods, used solvents, geographical area, etc. For example, in a comparative analysis, Sepahpour et al. [75] evaluated the variation of TFC in lemon grass using different solvent extraction systems. The author obtained 14.8 ± 0.5 in 80% acetone, 14.3 ± 0.1 in 80% ethanol, 11.7 ± 1.1 in 80% methanol, and 3.7 ± 0.1 in water (mg QE/g freeze-dried crude extract), which indicate significant difference of TFC depending on extraction solvent. In terms of quantity, our results are lower in all tested extracts and fractions, but the flavonoid’s quantity also varied depending on the type of extracts and fractions. In our case, optimum TFC was obtained in ME (fraction 29), which corresponds to curve 4 in graph b (Figure 3), followed by fraction 8 in aqueous extract, which corresponds to curve 1 in graph a (Figure 3), and in fraction 25 from the aqueous extract, which corresponds to curve 2, graph a in Figure 3. In comparison with other investigations, Godwin et al. [77] recorded values of total flavonoid concentration in *C. citratus* ranged from 6.9 to 11.3 μg/g quercetin equivalent (QE) and 6.9 to 12.9 μg/g QE dry weight basis for cold and hot percolations, respectively. Moreover, Unuigbe et al. [76] evaluated flavonoid contents in crude methanolic extract and its fractions (*n*-hexane, ethyl acetate, and chloroform) of powdered leaves of *C. citratus* using the Folin–Ciocalteu and aluminum chloride methods. The results revealed high flavonoid content in all tested extracts and their fractions. The ethyl acetate fraction had the highest flavonoid content (192.6 mg QE/g extract), followed by chloroform fraction (153.0 mg QE/g Extract), crude methanol extract (143.0 mg QE/g Extract) and fraction (*n*-hexane) (80.2 mg QE/g Extract). Currently, Mirzaei et al. [81] demonstrated that TFC quantity in lemon grass can be improved by 6% to 18% via plant growth-promoting rhizobacteria (PGPR) under water stress. These results showed the importance of climate conditions and interaction with beneficial microorganisms in influencing the biochemical contents of *C. citratus*.

### 2.6. Antioxidant Activity Determination

#### 2.6.1. Free-Radical FRAP Scavenging

Free-radical FRAP scavenging recorded in tested extracts of *C. citratus* is presented in Table 4. Obtained results showed a significant variation in scavenging activity depending on tested extracts and fractions. In the AE extract, free-radical FRAP scavenging was significantly variable among fractions. The highest value of FRAP was recorded in fractions 25 and 8. Similarly, in EE, the values were variable, and the highest value was recorded in fraction 26 compared to 36. In ME, values of scavenging activity were significantly variable, and the highest value was recorded in fraction 29, followed by fraction 23.

In a comparative study, Unuigbe et al. [76] measured the antioxidant capacity of crude methanolic extract of *C. citratus* and its *n*-hexane, chloroform, and ethyl acetate fractions. In results, *C. citratus* crude methanol extract exhibited promising antioxidant power with FRAP values of 157.55, 195.32, 212.02, and 243.91 mM ferrous sulfate equivalent per gram of extract for *n*-hexane, chloroform fraction, crude methanol extract and ethyl acetate fraction, respectively. Despite the difference in the recorded values in our study, the obtained result from this assay suggests that *C. citratus* extract may play a protective role against oxidative damage. In comparison with previous studies, Sepahpour et al. [75] recorded a wide range of FRAP values (mg QE/g freeze-dried sample extract) in *C. citratus* depending on solvent extraction systems;10.3 ± 1.4 for 80% acetone, 8.4 ± 1.0 for 80% ethanol, 9.8 ± 1.5 for 80% methanol, and 0.5 ± 0.1 for water. Similarly, in our case, the highest values of FRAP were obtained in fraction 29 of AE (97.89%) and fraction 25 of AE (75.86%). Equally, the quantity of flavonoids in fractions of each extract and among fractions is suggested to vary depending on the size of separated molecules in each extract and fraction, as mentioned by El Gharras et al. and Oracz et al. [79,82].

#### 2.6.2. Total Antioxidant Capacity (TAC)

The recorded total antioxidant capacity (TAC) in tested *C. citratus* extracts is presented in Table 5. Obtained results showed a significant variation of TAC depending on tested extracts and fractions. In AE, TAC was significantly variable among used fractions. TAC’s highest value was recorded in fraction 25, followed by fraction 8. Similarly, in EE, TAC values were variable, and the highest value was recorded in fraction 26 compared to fraction 36. In ME, TAC values were significantly variable. The highest value was recorded in fraction 29, followed by fraction 23.

Aourach et al. [83] compared the TAC of aqueous extracts of lemon grass, laurel, and cotton lavender using the DPPH scavenging activity test. TAC values (mg AAE/g dw) were as follows: 34.15 ± 0.59 for cotton-lavender, 21.80 ± 0.50 for Laure, and 9.54 ± 0.52 for lemon grass. In our case, the TAC value was superior in methanol and ethanol extracts when compared to water. Due to the importance of antioxidant capacity in *C. citratus*, the plant and its derivatives are used in nutrition and against pathogen microorganisms [84,85]. On the other hand, TAC values variation among fractions of each extract and extract of our study plant is suggested to be governed by the size of the separated molecule in each extract and fraction, as mentioned previously by Pisoschi and Negulescu [86].

#### 2.6.3. Dimensional Analysis of Antioxidant Activities and Fraction Content

**Figure 4:**
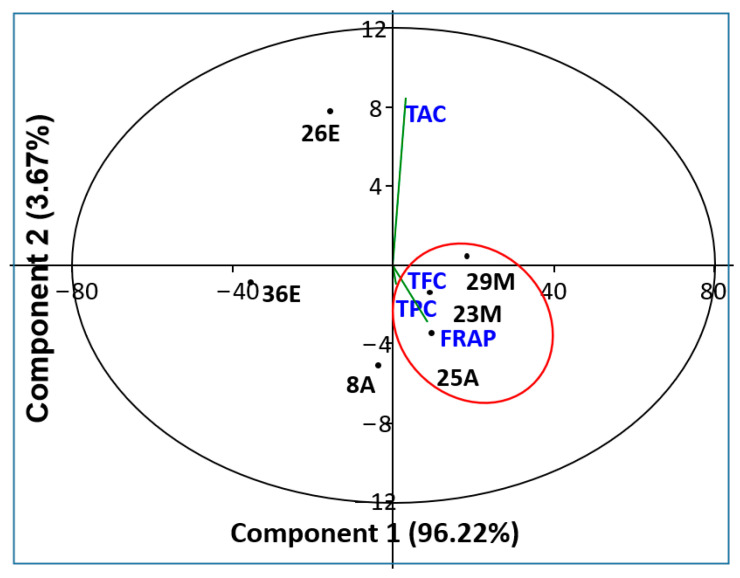
PCA plot (2D) of correlation between antioxidant Activities (FRAP and TAC) and TFC and TPC compounds in fractions of aqueous (A), methanol (M), and ethanol (E) extracts.

The relationship between antioxidant activities and chemical compounds, including TPC and TFC, are presented in Figure 4 and Table 6. Principal component analysis (PCA) showed that fractions 25 of AE and fractions 23 and 29 of ME demonstrated higher quantities of TPC and TFC. In contrast, fractions 26 and 36 of EE were characterized by lower TFC and TPC compounds. On the other hand, FRAP was correlated to TPC and TFC quantities in fraction 25 of aqueous and fractions 23 and 29 of ME. This relationship is confirmed by linear regression presented in Table 5. In contrast, TAC was not related to the quantity of TFC nor TPC in any fraction of tested extracts.

Previously, Wang et al. [87] investigated the relationship between chemical compounds counting TFC and TPC and both FRAP and DPPH antioxidant activities in *Ziziphus jujuba* Miller during three edible maturity stages. In the results, the authors demonstrated that FRAP was positively correlated with TFC and TPC compounds. Similar results were also mentioned in two cultivars of papaya fruit [88]. In our case, the correlation between FRAP, TPC, and TFC was noted only in fractions of aqueous and methanol extracts characterized by higher quantities of medium molecular size. This fact needs more investigations to clarify the relationship between the constituents of fractions and quantities of TFC and TPC than with their antioxidant activities.

The molecular weight of phenolic compounds identified by exclusion chromatography (on Sephadex gel) allowed the classification of phenolic compounds into monomeric, oligomeric, and polymeric forms [89]. Biological activities are strongly linked to the size of phenolic compounds [90,91]. Monomeric phenolic compounds are characterized by strong antimicrobial activity, and oligomeric and polymeric forms are characterized by antioxidant, anti-inflammatory, and even anti-cancer activity [92,93,94]. This analysis highlights the correlation between the molecular weight of the phenolic compounds and the identified biological activities. This is in perfect agreement with the results of our dimensional analysis (principal component analysis).

### 2.7. Antimicrobial Activity

The inhibitory effects of *C. citratus* extracts and their fractions against bacteria and fungi are presented in Table 7. The obtained results showed significant inhibitory activity in all tested extracts of *C. citratus* against both tested Gram-negative and positive bacteria and fungi. However, the minimum inhibitory concentration MIC and minimum bactericide concentration MBC were variable depending on the type of extract and tested microorganism. In AE, MIC values were similar for all tested bacteria, counting Gram-negative bacteria (*Escherichia coli* and *Pseudomonas aeruginosa*) and Gram-positive bacteria (*Bacillus cereus* and *Staphylococcus aureus*). In contrast, MIC values were significantly different between fungi, where the highest value was recorded against *Saccharomyces cerevisiae* when compared to *Candida tropicalis*. In addition, the MBC index was similar for all treated bacteria and fungi in aqueous extracts. In EE, MIC values were similar for all treated bacteria, including *Escherichia coli*, *Pseudomonas aeruginosa*, *Bacillus cereus*, and *Staphylococcus aureus*. On the other hand, the MIC values were significantly different between the two yeast strains tested. The highest value was recorded in *Saccharomyces cerevisiae* (non-pathogenic) compared to the pathogenic *Candida tropicalis* strain. In contrast, MBC values were similar for all treated bacteria and fungi in ethanol extracts. In ME, the MIC values were significantly different for all treated bacteria and fungi. In bacteria, the MIC was significantly superior against *Pseudomonas aeruginosa* and *Bacillus cereus*, followed by *Staphylococcus aureus*, and *Escherichia coli*, respectively. In fungi, MIC was significantly superior against *Candida tropicalis* compared to *Saccharomyces cerevisiae*. In contrast, MBC was similar between both treated fungi. However, the lowest MIC values mean higher inhibitory effects.

In this study, we introduced two antimicrobial molecules, namely Cefoxitin (antibiotic for bacteria) and Fluconazole (antifungal for pathogenic yeasts). The results show that Cefoxitin is very effective on *Escherichia Coli*, *Staphylococcus aureus*, and *Bacillus cereus* and has no effect on *Pseudomonas aeruginosa*, which is resistant to this antibiotic. However, this antibiotic remains slightly more effective compared to phenolic compounds. Phenolic compounds are more effective when compared to the antibiotic (Cefoxitin). This is the effect of the matrix formed by the mixture of phenolic compounds, which collaborate mutually to attenuate the *Pseudomonas aeruginosa* strain.

Concerning the three extracts’ antifungal activity, we note that the *Saccharomyces cerevisiae* strain is insensitive to Fluconazole but sensitive to the matrix of phenolic compounds of the three extracts. The *Candida tropicalis* strain is sensitive to Fluconazole at lower concentrations compared to phenolic compounds, which exert significant fungal activity. From this antimicrobial activity, we can conclude that the phenolic compounds of the three extracts are good candidates for preserving foodstuffs against microbial spoilage.

*C. citratus* is an important medicinal plant, and its bioactive molecules were widely tested against a wide range of microorganisms, including bacteria, fungi, and viruses [9,40,89,95]. For example, Balakrishnan et al. [65] tested *C. citratus* leaf extracts obtained serially by solvents of methanol, chloroform, and water against *Pseudomonas aeruginosa*, *Bacillus subtilis*, *Proteus vulgaris*, *Nocardia* sp., *Staphylococcus aureus*, *Serratia* sp., and *Enterobacter aeruginosa* microorganisms via the Kirby–Bauer agar disc diffusion technique. Results showed that *C. citratus* extracts exhibited maximum zones of inhibition in chloroform, methanol, and water extracts. Besides, *C. citratus* extracts exhibited a maximum zone of inhibition against *Bacillus subtilis*, *Pseudomonas aeruginosa*, and *Proteus vulgaris*. Analyzed data in the present work (the antibacterial activity of *C. citratus* plant (leaf extracts)) showed good results (inhibitory activity) for Gram-positive and Gram-negative microorganisms [65]. 

With the use of disc diffusion and vapor diffusion methods, the EO of *C. citratus* exhibited a promising antifungal effect against *Candida tropicalis*, *Aspergillus niger*, and *C. tropicalis* with different inhibition zone diameters [96]. Similarly, essential oils, mainly β-citral (neral) and α-citral (geranial), isolated from *C. citratus* leaf have been reported to be effective against *Campylobacter jejuni*, *Clostridium botulinum*, *Escherichia coli*, *Listeria monocytogenes* and *Salmonella* [28]. Moreover, Neetu Jain and Meenakshi Sharma [97] tested the inhibitory effects of EOs extracted from *C. citratus* leaves and their fractions against *Trichophyton mentagrophytes*, *T. rubrum*, *Microsporum canis*, *M. fulvum*, and *Candida tropicalis*. The results revealed a significant inhibitory effect against all tested microorganisms, and MIC ranged between 0.1 against *T. mentagrophytes*, *T. rubrum*, and *T. verrucosum*, and 0.5 against *C. tropicalis*.

In terms of extracts, PhangSiao Ze et al. [98] investigated the in vitro antimicrobial activities of extracts from the stem of *Cymbopogon citratus*. The experiment implicated successive extraction via the Soxhlet extraction approach using different solvents such as acetone, ethanol, dichloromethane, and methanol. The obtained results showed that dichloromethane extracts possess the highest inhibitory effect against *Acinetobacter baumanni*, *Escherichia coli*, *Neisseria gonorrhoeae*, *Bacillus cereus*, *Staphylococcus aureus*, and *Streptococcus pyogenes*. The inhibition zone varied between 2 and 14 mm. Moreover, Hassan et al. [26] evaluated the antimicrobial activity of methanol extract from lemon grass leaves. The inhibitory effect was tested against three bacterial strains *Staphylococcus aureus*, *Listeria* spp., and *Bacillus subtilis*. The findings demonstrated that at the maximum dose of 150 mg/mL methanol extract, the extract displayed a maximal zone of inhibition (35 mm) against *Bacillus subtilis*. Subramaniam et al. [99] investigated the inhibitory effect of essential oil, methanolic, and aqueous extracts of *C. citratus* against multidrug-resistant bacteria, including eight Gram-positive and eight Gram-negative strains. An agar well-diffusion assay was used for the methanolic and aqueous extracts. When compared to essential oil, both isolates were less sensitive to methanolic extracts because they generated zones of inhibition that were about three times smaller, whereas no zone of inhibition was created by the boiling extracts. Compared to the leaf or root extracts, all Gram-positive bacteria showed a higher susceptibility to the essential oil. Gram-positive bacteria showed comparable susceptibilities to leaf and root extracts. The pure essential oil demonstrated a one-fold larger zone of inhibition against *S. aureus*, demonstrating S. epidermidis’s superior efficacy against it. On the other hand, *C. citratus* leaf and root extracts worked better against *S. aureus* than *S. epidermidis*. The majority of Gram-negative bacteria examined showed similar zones of inhibition in *C. citratus* extracts, with nearly all of them being less susceptible to the methanolic extracts of the plant’s leaves and roots than to the essential oil. The mentioned antimicrobial activities of *C. citratus* extracts in the bibliography are similar to our findings, while those of essential oils were inferior. This is normal because the composition of essential oils and extracts are extremely different. However, this comparison shows that both essential oils and extracts have significant inhibitory effects against a wide range of pathogenic microorganisms.

The inhibitory effects of the tested extracts are suggested to be governed by their chemical compounds. Moreover, apigenin-7-o-rutinoside and luteolin-7-O-rutinoside recorded in methanol extracts are currently confirmed to have significant inhibitory effects against a wide range of microorganisms, including pathogens [100,101]. Similarly, kaempferol-3-O-glucuronide recorded in water extracts is known for antimicrobial activities [102]. For example, an aqueous extract of *Stachys parviflora* containing Apigenine-7-o-rutinoside showed a significant inhibitory effect against *Xanthomonas campestris*. These molecules can pierce the membrane and internal compounds of microorganisms either with single effects or with synergetic actions among a group of bio-compounds [103,104,105]. On the other hand, the variation in MIC and MBC among fractions of each extract and between the extracts is suggested to be governed by the size of separated molecules in each extract as well as the variation in TPC and antioxidant activities. Equally, the resistance of each tested bacteria and fungi is suggested to influence the inhibitory effect of each tested extract and fraction.

## 3. Materials and Methods

### 3.1. Plant Material

In this study, we used lemon grass leaves cultivated in the botanical garden belonging to the Sciences and Technologies Faculty in Fez (Central Morocco). Samples were collected in April 2022 and taken to the Functional Ecology and Environment Engineering laboratory for further investigations. Weighted sample leaves were dried under ambient conditions, powdered (moisture 9 ± 05%), filtered (0.01 mm), and preserved until use.

### 3.2. Crude Extracts Preparation

Three samples of plant powder (10 g) were mixed with 200 mL of solvents (water, methanol, and ethanol). Extraction was carried out three times by maceration for two hours at room temperature. Collected liquid extracts (600 mL) were filtered and dried (rotavapor) to acquire a dry residue.

### 3.3. Chemical Compounds Analysis

To identify the extract’s chemical compounds, we used liquid chromatography coupled with mass spectrometry, abbreviated LC-MS-MS. In our case, 1 μL of each extract was injected onto a reverse phase C18 type column (ACQUITY UPLC-BEH C18, particle size 1.7 μm), with dimensions equal to 2.1 mm × 100 mm. The mobile phase consists of two eluents: H_2_O + 0.1% Formic acid (FA) and acetonitrile+0.1% FA. Seal Wash Period: 5 min. High Pressure Limit: 18,000 psi. Flow Rate 0.5 mL/min. Acquisition time: 15 min. Function Mode: High Definition MS^E^; low mass: 50 *m*/*z*; high mass: 1000 *m*/*z*; scan time: 0.250 s.

### 3.4. Total Phenolic Compounds Determination

To determine the total phenolic compound content, the Folin–Ciocalteu method was modified as stated by Singleton et al., (1999) [106]. Further, 50 µL of each extract and its fractions was mixed for five minutes with 450 µL of 0.2 N Folin–Ciocalteu reagent then 450 µL of the 75 g L^−1^ Na_2_CO_3_ solution was added. After being at room temperature in the dark for two hours, each sample absorbance was then measured at 760 nm (Shimadzu UV-1600 PC UV spectrophotometer). Standard calibration curves (y = 1.6021X + 0.0683, R^2^ = 0.99) were done by concentrations ranging from 0.008 to 1 mg/mL in gallic acid ethanolic solution. Triplicated experiment results are expressed as mg gallic acid equivalents (GAE) per g of dried plant material (mg GAE/).

### 3.5. Gel Filtration Chromatography

The aqueous phenolic extract of lemon grass is a mixture of monomeric, oligomeric, and polymeric forms; it is essential, according to the objectives of this work, to originate their separation. The fractionation method is a size-exclusion chromatography allowing molecules to separate according to their molecular size. Sephadex gel is composed of highly porous microbeads, where molecules with the highest molecular weights diffuse only outside the pore beads and exit the column first. On the other hand, small phenolic compounds diffuse inside all the microbeads, are delayed, and then exit the column.

A column with a diameter of 2.5 cm and a length of 50 cm was used with a flow rate set at 1 mL/min, based on the method of Siddiqui et al. with some modifications [107]. An amount of 20 g of Sephadex G50 was mixed with 150 mL of lithium chloride buffer solution (5 mM NaOH, 2.5 mM LiCl). Further, 0.5 mg/mL of each extract was fractionated on Sephadex gel, and the separated fractions were collected in test tubes at a volume of 2 mL and analyzed with a spectrophotometer at 380 nm for phenolic compounds [108].

### 3.6. Total Flavonoid Content (TFC) Determination

Total flavonoids were estimated using the method of Woisky and Salatino (1998) [109]. To 0.5 mL of sample (fractions of each extract), 0.5 mL of 2% AlCl_3_ ethanol solution was added. After 1 h at room temperature, absorbance was measured at 420 nm. Total flavonoid contents were expressed as quercetin equivalent from a calibration curve as mg of quercetin equivalent (QE) to a gram of the sample’s dry weight (mg QE/g).

### 3.7. Antioxidant Activity Determination

#### 3.7.1. Ferric Reducing-Antioxidant Power (FRAP)

To evaluate antioxidant activity in *C. citratus* extracts and fractions, we used the protocol described by Aazza [110]. First, each prepared sample or standard was mixed with phosphate buffer (2.5 mL, 0.2 M, pH 6.6) and potassium ferricyanide [K_3_Fe(CN)_6_] (2.5 mL, 1%). The mixture was incubated at 50 °C for 20 min. A portion (2.5 mL) of trichloroacetic acid (10%) was added to the mixture, which was then centrifuged for 10 min at 3000× *g*. The upper layer solution (2.5 mL) was mixed with distilled water (2.5 mL) and FeCl_3_ (0.5 mL, 0.1%), and absorbance was measured at 700 nm in a spectrophotometer. In our case, we recovered the fractions and regrouped them into the following groups: aqueous fractions 8 and 25; methanolic fractions 29 and 45; and ethanolic fractions 26 and 36.

#### 3.7.2. Total Antioxidant Capacity (TAC)

According to Libbey and Walradt [111], green phosphomolybdenum complex production measured the total antioxidant activity (TAC) of all generated samples (extracts and their fractions). In Falcon 15 mL tubes, a 25 μL aliquot of sample solution was mixed with 1 mL of reagent solution (0.6 M sulfuric acid, 28 M sodium phosphate, and 4 M ammonium molybdate). After that, the Falcon tubes were incubated at 95 °C for 90 minutes. The mixture absorbance was measured at 695 nm against a blank. The calibration curve was done by aqueous ascorbic acid solution (y = 0.7889x + 0.0492, R^2^ = 0.996) in concentrations ranging from 0.0039 to 5.000 mg/mL. The experiment was carried out in triplicate, and the antioxidant activity results are mean values given as g of ascorbic acid equivalents (AAE) g^−1^ of dried plant material.

### 3.8. Antimicrobial Activity

The antimicrobial activity was quantitatively (by microdilution), carried out by minimum inhibitory concentration (MIC) evaluation on three microbial models, namely two Gram-negative bacteria (*Pseudomonas aeruginosa* ATCC27653 and *Escherichia coli* CIP5412), two bacteria Gram-positive (*Staphylococcus aureus* CIP543154 and *Bacillus cereus* ILP1428B) and two fungal strains (*Candida tropicalis* Y1512 and *Saccharomyces cerevisiae* YMES2). These microorganisms were targeted because they represent biological models of prokaryotes and prokaryotes on the one hand; on the other hand, they are agents of potential pathogenicity for humans.

A volume of 50 μL of bacterial broth, diluted to 10^6^ cells/mL using Luria–Bertani (LB) medium, and 50 μL of different concentrations of extracts (3.90–1000 µg/mL) were added in a 96-well microtiter plate, respectively, and further incubated for 20 h at 37 °C. The mixture of 50 μL bacterial solution and 50 μL of sterile medium was used as a positive control. After incubation time, 15 μL of resazurin (0.015%) was put into wells and then incubated for 2 h to visualize color changes. Minimum bactericidal concentration (MBC) was obtained according to Barry et al. (1987) (modified); the contents of wells containing ½ × LIC, LIC, 2 × LIC, and 4 × LIC were transferred onto agar plates and further incubated at 37 °C for 24 h. A similar approach was applied to fungi strains in YPG (10 g of Yeast extract, 20 Bactopepetone, 10 g of Glucose in water (1l)).

As a control, we used two molecules with antimicrobial (Cefoxitin) and antifungal (Fluconazole) activity, respectively.

### 3.9. Statistical Analysis

Each extract was treated as a single treatment for statistical analysis purposes. Three independent measurements were made for each. We calculated means, and the results were presented as means ± SD. All studied parameters, including TPC, TFC, TAC, and FRAP, were tested for normality and homogeneity of variance. Further, TPC, TFC, TAC, and FRAP in fractions of aqueous extracts, as well as TAC and FRAP in fractions of methanol extracts, were compared with Analysis of Variance (ANOVA) one-way tests (for three or more variables). In parallel, TPC, TFC, TAC, and FRAP in fractions of ethanol extracts were compared with the results of *t*-tests (two variables). The deconvolution method was carried out with OriginLab 8 Software using multi-peaks fitting with Gaussian peak type [112]. To test the correlation between compounds (TFC and TPC) and antioxidant activities in fractions of used extracts, we used Principal Component Analysis (PCA). Equally, FRAP and TAC antioxidant activity predictors were tested with multiple linear regression, in which TPC and TFC were considered independent variables. All tests were realized in STATGRAPHICS centurion XII.

## 4. Conclusions

This study investigated bioactive molecules, including phenolic compounds (TPC), flavonoids (TFC), and antioxidant activity (TAC and FRAP) in various extraction solvents and fractions of lemon grass cultivated in Morocco. Equally, we tested their inhibition capacity against bacteria and fungi. The studied extracts showed different bioactive molecules characterized by various molecular weights based on Sephadex and identified by LC-MS/MS analysis. The highest diversity of bioactive molecules (i.e., apigenin-7-O-rutinoside and myricitin-3-O-rutinoside) was recorded in methanol solvents (n = 8), followed by water extract with five molecules (i.e., kaempferol-3-O-glucuronide) and ethanol with three molecules (i.e., quercetin-3-O-rutinoside). 

Further, all extracts and their fractions showed significant and variable quantities of TPC and TFC. The highest value of TPC was obtained in fraction 25 of AE, and the highest value of TFC was obtained in fraction 29 of ME. Generally, high TPC and TFC correlated with high DPPH and FRAP values, indicating that phenolic compounds were mainly responsible for extracts’ antioxidant activities. The correlation between FRAP TPC and TFC was noted only in fractions of AE and ME characterized by a higher quantity of medium molecular size. The highest FRAP radical scavenging activity was obtained in fraction 29 of ME and the highest total antioxidant capacity (TAC) was obtained in fraction 29 of ME. All tested extracts of *C. citratus* and their fractions showed significant inhibitory activity against both tested bacteria and fungi. The biological activity of the methanolic extract is explained by the presence of a higher number of active molecules. This study presented new data on bioactive molecules in the extracts of cultivated *C. citratus* in the Moroccan environment. This analysis highlights the correlation between the molecular weight of the phenolic compounds and the identified biological activities. Equally, throughout this research, we related the active molecules and biological activities of the plant, which is suggested to promote the use of the plant in food and remedies. However, pharmacological studies are needed to evaluate the effects of lemongrass extracts on human health. This study also suggests that the extraction solvent and molecular size can affect the phytochemical profile and the antioxidant activity of these extracts, which is suggested to affect their pharmaceutical uses. Equally, the toxicity of the recorded bioactive molecules must be tested before any remedy is used in humans.

## Figures and Tables

**Table 1:** Chemical compounds identified in the extracts of lemon grass.

Extract	Compound	Component Name	RT	*m*/*z*
Methanol	1	Kaempferol-3-O-rhamnoside	9.95	417.21
	2	11beta,17alpha,21-Trihydroxy-4-pregnene-3,20-dione 21-caprylate	5.70	487.31
	3	Quercetin-3-O-arabinoside	11.12	379.16
	4	Luteoline-7-O-rutinoside	11.19	393.22
	5	Digalactosyl diglyceride	11.03	457.19
	6	Quercetin-3-O-glucuronide	10.61	431.23
	7	Apigenin-7-O-rutinoside	5.70	585.20
	8	Myricitin-3-O-rutinoside	10.71	565.21
Water	1	Kaempferol-3-O-glucuronide	5.70	487.30
	2	Quercetin-3-O-galactoside	0.27	477.08
	3	4-pregnene-11beta,17alpha,21-triol-3,20-dione 21-caprylate	6.06	487.30
	4	Kaempferol 3-O-rutinoside	11.03	457.19
	5	Myricetin	0.27	377.08
Ethanol	1	Apigenin-7-O-glucuronide	3.06	545.13
	2	Quercetin-3-O-rutinoside	11.03	665.17
	3	Myricetin-3-O-glucuronide	6.16	497.33

**Table 2:** Evaluation of total polyphenol content (TPC) depending on extracts and fractions (denote statistically * < ** < *** < ****; * equivalent to *p* < 0.05).

Extracts	Fractions	TPC [mg/g]
*Aqueous extract* (AE)	8	2.04 ± 0.11 **
	16	2.77 ± 0.12 ***
	25	4.60 ± 0.29 ****
	58	0.33 ± 0.05 **
	75	0.11 ± 0.03 *
*Ethanolic extract* (EE)	26	0.89 ± 0.09 **
	36	0.11 ± 0.08 *
*Methanolic extract* (ME)	23	2.83 ± 0.80 *
	29	3.26 ± 0.44 **

**Table 3:** Total flavonoid content (TFC) evaluation depends on extracts and fractions (denote statistically * < ** < ***; * equivalent to *p* < 0.05).

Extracts	Fractions	TFC [mg QE/g]
*Aqueous extract* (AE)	8	0.60 ± 0.07 ***
	16	0.46 ± 0.07 *
	25	0.57 ± 0.07 **
*Ethanolic extract* (EE)	26	0.26 ± 0.03 **
	36	0.19 ± 0.05 *
*Methanolic extract* (ME)	23	0.48 ± 0.12 *
	29	0.70 ± 0.08 **

**Table 4:** Free-radical FRAP scavenging in *C. citratus* depending on extracts and fractions.

Extracts	Fractions	FRAP (%)
*Aqueous extract* (AE)	8	63.91
	25	75.86
*Ethanolic extract* (EE)	26	48.53
	36	32.67
*Methanolic extract* (ME)	23	75.00
	29	97.89

**Table 5:** Total antioxidant capacity (TAC) evaluation in *C. citratus* depending on extracts and fractions.

Extracts	Fractions	TAC
*Aqueous extract* (AE)	8	68.98%
	25	75.18%
*Ethanolic extract* (EE)	26	77.21%
	36	62.35%
*Methanolic extract* (ME)	23	76.81%
	29	89.89%

**Table 6:** Antioxidant activity predictors in fractions of tested extract analyzed with simple regression.

Antioxidant Activity	Predictors	SS	Df	MS	F-Ratio	*p*-Value
FRAP	TPC	9.39	1	9.39	9.33	0.03
	Residual	4.03	4	1.01		
	TFC	0.16	1	0.16	19.23	0.01
	Residual	0.03	4	0.01		
TAC	TPC	4.14	1	4.14	1.78	0.25
	Residual	9.28	4	2.32		
	TFC	0.07	1	0.07	2.30	0.20
	Residual	0.13	4	0.03		

SS—sum of squares; Df—degree of freedom MS—mean square.

**Table 7:** Comparison of minimum inhibitory concentration (MIC) (µg/mL) and minimum bactericide or fungicide concentration (MBC/MFC).

Taxon		Tested Strains	AE		EE		ME		Cefoxitin		Fluconazole	
			MIC	MBC	MIC	MBC	MIC	MBC	MIC	MBC	MIC	MFC
Bacteria	Gram −	*Escherichia coli*	6.25	12.25	6.25	12.25	3.12	12.25	1.56	3.12		
		*Pseudomonas aeruginosa*	6.25	12.25	6.25	12.25	12.25	12.25	R	R		
	Gram +	*Bacillus cereus*	6.25	12.25	6.25	12.25	12.25	12.25	3.12	3.12		
		*Staphylococcus aureus*	6.25	12.25	6.25	12.25	6.25	12.25	1.56	3.12		
Fungi		*Candida tropicalis*	12.25	12.25	12.25	12.25	12.25	12.25			1.56	3.12
		*Saccharomyces cerevisiae*	6.25	12.25	6.25	12.25	6.25	12.25			R	R

AE—aqueous extract; EE—ethanolic extract; ME—methanolic extract and R—resistance.

## Data Availability

The datasets used and analyzed during the current study are available from the corresponding author upon reasonable request.
